# Study on the influence of customers’ consumption intention in online food delivery service: Calorie label as the moderator variable

**DOI:** 10.1371/journal.pone.0326617

**Published:** 2025-06-26

**Authors:** Huilin Yuan, Jue Wang

**Affiliations:** 1 College of Economics and Management, Jiangsu University of Science and Technology, Zhenjiang, China; 2 School of Finance & Economics, Wuxi Institute of Technology, Wuxi, China; University of Georgia, UNITED STATES OF AMERICA

## Abstract

Online food delivery (OFD) service, is a catering service model taking the Internet as the platform, where customers place orders online and enjoy offline delivery services. Customers’ attention on calorie labels when ordering food online is increasing with the increased health awareness in recent years. According to SERVQUAL model and Theory of Planned Behavior, this article constructed a structural equation model and then introduced calorie labels as a moderator variable. The data was obtained through a questionnaire survey, and a total of 222 valid responses from Chinese OFD service users were collected. Data was measured by SPSS software, and the structural equation model was analyzed by AMOS software. The results showed that food quality (path coefficient = 0.652, p < 0.001) and the kitchen photos (path coefficient = 0.849, p < 0.001) had greater influence on customers’ consumption intention. At the same time, calorie labels for OFD service stores have partly moderating effect on customers’ consumption intention. Regarding light-meal food and home-cooked food stores, calorie labels have a significantly positive moderating effect on the impact of OFD service quality on customers’ consumption intention. Regarding dessert and fried food stores, calorie labels have no moderating effect. The above researches put forward model reference and decision-making reference for improving OFD service quality and customers’ consumption intention and also contribute to the literature on OFD service by offering new insights into calorie labels.

## 1 Introduction

The 54th Statistical Report on the Development of the Internet in China showed that the number of OFD service users in China accounts for 50.3% of the total scale of Internet users [1]. The OFD service industry is an extension and expansion of the traditional catering industry through the Internet platform [2]. Unlike the traditional catering industry, OFD service customers cannot perceive the store environment and observe the process of food production. Also, they failed to experience the dining service in the store. Even more, they rarely get direct feedback from other consumers. Customers can only feel a finished product, which brings some uncertain factors [3].

To quantify the impact of these uncertain factors on customers’ consumption intention, a series of effective studies were carried out. Chen et al. [4] studied the relationship between attitudes toward delivery platform service and consumers’ purchase intention, which showed that negative attitudes towards platform service would hurt consumers’ purchase intention. Namkung et al. [5] studied the relationship between restaurant food quality, customer satisfaction, and behavior. The results showed that the overall quality, taste, and appearance of food significantly affects customer satisfaction. Chen et al. [6] used eye tracking to analyze how online product reviews influence consumers’ purchasing decisions. It highlighted those consumers, especially women, pay more attention to negative reviews, which affected their purchase intentions. Liu et al. [7] took Chinese consumers as research objects and found that Chinese consumers are willing to pay a premium for safer food. The above research indicated that the rapid growth of the industry was accompanied by several critical factors influencing consumer purchasing decisions, which include prices, packaging, delivery speed, freshness of ingredients, comments, transparency of kitchen operations and hygiene practices.

In terms of research methods, we decided to use Structural Equation Modeling (SEM). SEM is a multivariate data analysis method for analyzing complex relationships among constructs and indicators [8], and many researchers use it to study consumption intention, satisfaction, trust, and other dependent variables. Zhao et al. [9] used SEM to study electric word of mouth and consumers’ purchase intention in social e-commerce. Hanaysha [10] used SEM to study the influence of social media marketing characteristics on consumers’ purchase decisions in the fast-food industry where brand trust acts as an intermediary. Wongpitch [11] used SEM to study the impact of corporate social responsibility motivation on the purchase intention model. Sriram [12] used SEM to study the impact of social media advertising on consumers’ purchase intention. Wang et al. [13] used SEM to study the impact of consumer perception on purchase intention based on cross-business e-commerce platforms. Wang et al. [14] used SEM to study the impact of food quality, service quality, and price on customer trust in organic food sales. Hong et al. [15] applied SEM to the influence of customer perceived value on customer satisfaction and loyalty in food delivery robot service. These studies collectively demonstrated that consumers’ consumption intention, trust, and satisfaction were influenced by a variety of factors, including social media marketing, food quality, service quality, perceived value and others. As a powerful analytical tool, SEM has enabled researchers to uncover the relationships among these factors and provided theoretical support for enterprises.

Nowadays, the calories of food are also critical factors influencing consumers’ willingness to purchase. “Kilocalorie” is the unit measurement of calories in nutrition. The International Food and Health Institute’s 2022 Food and Health Survey Report [16] discussed the trend of public concern about food labels, including calorie content. The report also indicated that more consumers paid attention to labels in 2022 compared to 2021, and 52% of consumers always paid attention to labels when shopping online. According to the 2020 Ipsos poll, people tended to choose a “calorie in, calorie out” approach, indicating that they tried to burn more calories than they took in to stay healthy. A healthy lifestyle had gradually shifted from merely meeting basic food needs to prioritizing nutrition, balance, and health. At the same time, according to Cognitive Market Research [17], the global calorie counter websites and apps market size were USD 1751.5 million in 2024, and it will expand at a compound annual growth rate (CAGR) of 13.50% from 2024 to 2031. From this, we can know that people’s attention to calorie labels has been increasing year by year. However, because kinds of OFD service stores have different target groups and different food products, the impact of calorie labels on consumer consumption intention may play a different role. Therefore, this article will study the impact of OFD service quality factors on consumers’ consumption intention, as well as the moderating effect of calorie labels to different stores.

## 2 Assumptions and models

### 2.1 OFD service quality factors

According to the SERVQUAL model proposed by Parasuraman et al., service quality is determined by five dimensions: Tangibility (such as the application interface of the OFD service platform, OFD service packaging, delivery personnel equipment, etc.), reliability (such as the timeliness of OFD services, the guarantee of food quality, etc.), responsiveness (such as the response speed of customer service, the efficiency of solving problems, etc.), assurance (such as payment safety, food safety, etc.), empathy (such as personalized OFD services, etc.). In addition, the Theory of Planned Behavior (TPB) can further explain the relationship between service quality and consumption intention. According to TPB, an individual’s intention to perform a behavior is influenced by three factors: attitude, subjective norms, and perceived behavioral control. In the context of OFD service, a perfect performance of food quality, overall platform performance and so on will increase consumers’ consumption intention, whereas the opposite is true when these factors are bad.

Based on these, this study summarized the OFD service quality that affected the consumption intention of customers into five dimensions: food quality, comments, kitchen photos, delivery speed, and packaging. Among these, food quality is the core element. If the food quality is poor, customers will be dissatisfied, leading to negative comments and reducing the number of potential customers. Food quality is also influenced by delivery speed and packaging. Timely delivery ensures the temperature and freshness of the food, while high-quality packaging enhances the perceived value of the food, thereby strengthening the overall service experience. Additionally, kitchen photos refer to images of the cooking environment provided by vendors on OFD platforms. The inclusion of kitchen photos helps build customer trust in food hygiene and safety, further reinforcing the positive correlation between service quality and purchase intention. In summary, these variables are interconnected and collectively contribute to significantly enhancing customers’ consumption intentions.

Based on these, this article put forward the hypothesis of OFD service quality and customer consumption intention:

H1: OFD service quality positively promotes the consumption intention.

### 2.2 The moderating role of calorie labels

A study supported by the Centers for Disease Control and Prevention (CDC) [18] showed that calorie labels on menus helped consumers make healthier choices, thereby increasing their interest in healthy foods; Bawazeer et al. [19] studied how calorie labels on restaurant menus influenced students’ food choices; Heiman and Lowengart [20] found that calorie labels can affect people’s perception, and selection process of food; Amada et al. [21] found that people will use calorie labels to choose healthier foods to reduce disease; Natasha et al. [22]found that adults exposed to calorie labels would select 11 kcal less (equivalent to a 1.8% reduction), and consume 35 kcal less (equivalent to a 5.9% reduction). The research study by Tanasache et al [23]. showed that providing calorie information for individual items and for the entire order increases the probability of choosing a low-calorie main course among those aged 55 and older. In conclusion, calorie labels can change people’s food choices, so calorie labels can be used as an effective moderator variable. At the same time, because OFD service stores have varying market positioning and target customer groups, calorie labels may have different effects on them.

Based on this, the following hypothesis was proposed:

H2: Calorie labels have a moderating effect on the influence of OFD service quality on customers’ consumption intention.

### 2.3 Questionnaire and model

Lin et al. [24] found that high-quality dishes played a key role in driving consumers’ buyback intentions for specific stores. Riaz et al. [25] explored the impact of online reviews on users’ cognitive and emotional experiences are confirmed that positive reviews help encourage users to make repeat purchases. Ganesh et al. [26] used empirical data from cloud kitchens in India to analyze the impact of the delivery time on customer ratings. The findings also represented that delay significantly reduced the probability of positive evaluation, reinforcing the importance of timely delivery. Su and Wang [27] found that marketers often manipulated the outer packaging to influence consumers’ expectations, experiences, and behaviors. By evaluating food safety standards, Wei [28] emphasized that transparency in food hygiene practices would increase customer trust. Through these above researches, we can learn that food quality, comments, delivery speed, packaging, and kitchen’s hygiene are all important factors affecting the consumption intention of OFD service customers. Therefore, these factors will be used as sub-variables of OFD service quality and reflected in the following questionnaire design and model construction.

Based on the above references, the questionnaire design includes three parts: the first part is the basic information statistics of the surveyors. The second part consists of a survey on the consumption intentions of OFD service customers, as well as an investigation into their perceptions of food quality, comments, kitchen photos, delivery speed, and packaging. The third part focuses on consumers’ expectations and attitudes toward calorie labels in specific OFD service stores. The second and third sections employ a five-point Likert scale for evaluation and analysis, facilitating the quantification of consumers’ preferences and attitudes, thereby ensuring the reliability and validity of the research findings.

**Fig 1:**
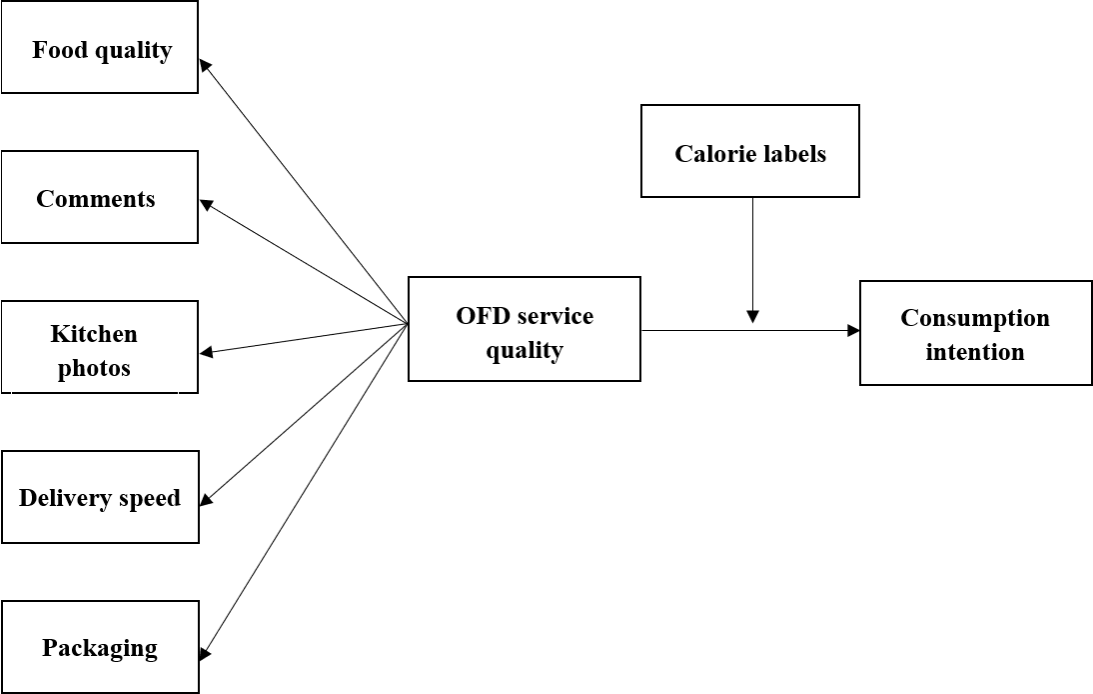
Model of the influence of calorie labels on consumers’ consumption intention.

Based on the above theoretical hypotheses and literature references, a structural equation model framework can be constructed: OFD service quality is taken as an independent variable, and a series of OFD service quality factors (food quality, comments, kitchen photos, delivery speed and packaging) are introduced as second-order latent variables; customers’ consumption intention is taken as the dependent variable, and calorie labels of various stores are introduced as the moderator variable. The model of the influence of calorie labels on consumers’ consumption intention is shown in Fig 1.

## 3 Study design

### 3.1 Data collection

To verify the influence of various factors on the consumption intention of OFD service customers, convenience sampling method was used to distribute the questionnaire through an online platform. Prior to the main collect data, we conducted a pilot test to evaluate the validity and reliability of the questionnaire. The pilot test involved a sample of 30 participants who were asked to provide feedback after completing a questionnaire, including whether the questions were clear and easy to understand. Based on the feedback, we revised the wording of some questions and removed the redundancy question. In addition, we also conducted a preliminary reliability and validity analysis, and the results showed that the questionnaire can effectively measure the target.

After conducting the pilot test, we began formal data collection through an online platform. A total of 269 questionnaires were collected, 222 of which were valid, with an effective questionnaire rate of 82.5%. The sample size refers to papers in similar fields or research methods. For example, Maryam et al. [29] collected 220 valid questionnaires to study customers’ purchase intention of home energy management system; Dewi et al. [30] researched the mediating roles of green work environments and engagement in Jakarta’s logistics sector through 222 questionnaires. Therefore, the sample size of 222 questionnaires for this study is reasonable, which can provide reliable data to support for the research conclusion.

Table 1 shows the basic characteristics of the respondents. The proportion of male and female respondents is 43.69% and 56.31% respectively, which indicated the gender distribution is relatively balanced. In terms of age, the survey targets are mainly young people aged 18–35, and the age distribution of the sample is consistent with the distribution of people who always order food online [31], which can effectively reflect the whole group of people who order food online. More than 70% of the respondents are students. The student population, as a representative group of young and trend-conscious individuals, ensures that the responses collected in the questionnaire reflect contemporary trends. At the same time, Li and Qin [32] find that the college students are the main group of OFD service, and they know this service well. Therefore, students can guarantee the quality of the questionnaire. The monthly income of the respondents is mainly 1000–2000 yuan, and the average unit price of OFD service order is mainly between 10–29 yuan [33]. Therefore, the overall economic situation can support the ordering behavior. Additionally, the proportion of respondents with a high frequency of ordering food online and a strong focus on calorie labels both exceeded half, indicating the validity of these two parts’ questionnaire responses. Sample feature distribution is shown in Table 1.

**Table 1:** Sample feature distribution (N = 222).

Basic information		Samples	Percentage (%)
Gender	Male	97	43.69
	Female	125	56.31
Age	18–25	174	78.38
	25–35	26	11.71
	35–45	18	8.11
	Over 45	4	1.80
Occupation	Student	162	72.97
	Salaryman	39	17.57
	Freelancer	11	4.95
	Other	10	4.51
Monthly income	0–1000	61	27.48
	1000–2000	82	36.94
	2000–4000	41	18.47
	Over 4000	38	17.12
Ordering frequency	Everyday	30	13.51
	2 to 5 times a week	102	45.95
	2 to 5 times a month	64	28.83
	Little	26	11.71
Attention to calorie labels	Highly concerned	26	15.71
	Moderately concerned	52	37.42
	Generally concerned	53	29.87
	Slightly concerned	65	11.29
	Unconcerned	26	5.71

### 3.2 Reliability

In this questionnaire, the main influencing factors were measured in the form of a scale. Reliability reflects whether the questionnaire is reliable. This article used Cronbach’s α coefficient to test the reliability of the questionnaire. The value of α ranges from 0 to 1. A larger value indicates that the questionnaire is more consistent and reliable. Generally speaking, “α” greater than 0.7 indicates a high reliability of the questionnaire, and “α” greater than 0.6 indicates that the data is acceptable.

This article used SPSS29.0 software to test the reliability of the questionnaire data. The overall reliability of the questionnaire is over 0.75. Among them, the reliability of the five dimensions of OFD service quality also exceeds 0.6, indicating that its internal consistency is good and the data is relatively reliable. The results are shown in Table 2, which represent the overall Cronbach’s α coefficient.

**Table 2:** Overall Cronbach’s α coefficient.

Cronbach’s α	Cronbach’s α based on standardized terms	Number
0.794	0.799	30

### 3.3 Validity

In order to ensure the validity of the measurement tool, we first conducted the model fit testing, and the test results were shown in Table 3. According to the results in Table 3, the Chi-square degree of freedom ratio is 0.517, which is within the range of 1–3. The root mean squared error is 0.056, which is within a good range. In addition, ITI (Incremental Fit Index), TLI (Tucker-Lewis index) and CFI (Comparative Fit Index) all reached a good level above 0.8. Therefore, the analysis results show that the model has a good construct validity.

**Table 3:** Model fit testing.

Index	Standard	Result
CMIN/DF	1-3 excellent,3–5 good	2.517
RMR	<0.05 excellent, < 0.8 good	0.056
IFI	>0.9 excellent, > 0.8 good	0.891
TLI	>0.9 excellent, > 0.8 good	0.853
CFI	>0.9 excellent, > 0.8 good	0.888

Under the premise of good construct validity of the model, the convergent validity and discriminant validity of each dimension of the scale will be further examined, typically using the Average Variance Extracted (AVE) and the square root of the AVE to assess these forms of validity. AVE value greater than 0.4 is considered acceptable, and greater than 0.5 is considered ideal [34]. In this study, the AVE values of the five latent variables range from 0.44 to 0.61, indicating good convergent validity of the model. Regarding discriminant validity, the standardized correlations between each pair of dimensions are all less than the square root of the corresponding AVE values, suggesting that the dimensions exhibit good discriminant validity. The results of each dimension’s discriminant validity are shown in Table 4.

**Table 4:** The results of each dimension’s discriminant validity.

	Food quality	Comments	Kitchen photos	Delivery speed	Packaging
Food quality	**0.488**				
Comments	0.294	**0.555**			
Kitchen photos	0.470	0.230	**0.442**		
Delivery speed	0.653	0.205	0.379	**0.447**	
Packaging	0.016	0.261	0.515	0.097	**0.609**
Square root of the AVE value	0.699	0.745	0.665	0.669	0.780

## 4 Structural equation model testing

### 4.1 Fit model analysis and multicollinearity analysis

Before conducting the formal analysis of the model, we first performed a fit model test and a multicollinearity analysis. The fit model test is a method used to evaluate whether the model is suitable for the data, while the multicollinearity analysis is employed to detect and eliminate the effects of multicollinearity, thereby ensuring the accuracy and reliability of the model.

As shown in Table 5, fitting indexes were calculated, and all of them reached satisfactory levels, indicating that the model is well-fitting and acceptable for further analysis.

**Table 5:** Fit Model testing.

Index	Standard	Result
CMIN/DF	1-3 excellent,3–5 good	2.436
RMR	<0.05 excellent, < 0.8 good	0.066
IFI	>0.9 excellent, > 0.8 good	0.864
TLI	>0.9 excellent, > 0.8 good	0.836
CFI	>0.9 excellent, > 0.8 good	0.862

To examine whether multicollinearity exists among the independent variables, we obtained the correlation coefficients of each independent variable by running the model, which was shown in Table 6. According to the book authored by Barbara and Linda [35] we know that multicollinearity won’t exist when the correlation between variables is less than 0.8. As shown in Table 6, all correlation coefficients are below 0.8, indicating that there is no multicollinearity among the independent variables.

**Table 6:** Correlations.

	Packaging	Delivery speed	Kitchen Photos	Comments	Food quality
Packaging	1				
Delivery speed	0.229	1			
Kitchen photos	0.310	0.533	1		
Comments	0.140	0.241	0.325	1	
Food quality	0.238	0.409	0.553	0.250	1

### 4.2 Hypothesis testing of customers’ consumption intention

**Fig 2:**
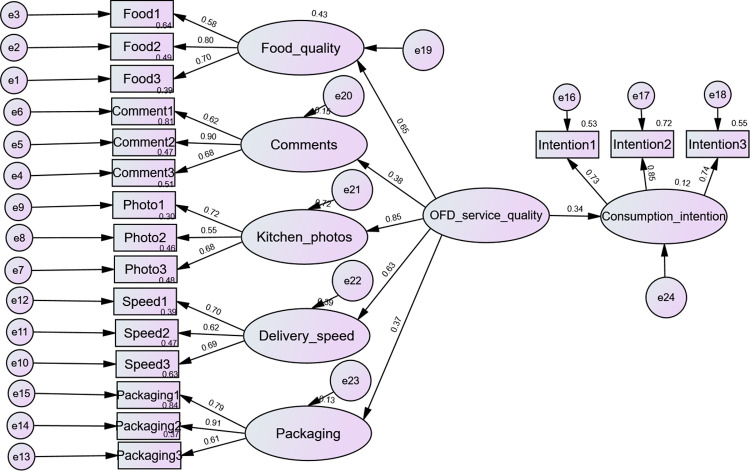
The model of OFD service quality factor, OFD service quality, and consumption intention.

Based on the reliability test and validity test of the questionnaire data, a model of the impact of OFD service quality on customers’ consumption intention was constructed, the observed variables of each latent variable were abbreviated as food, comment, photo, delivery speed and packaging respectively. Subsequently, Amos28.0 software was used to obtain the path coefficients of the model, which is shown in Fig 2, and then the hypothesis test results of the model were integrated, as shown in Table 7.

**Table 7:** Significant relationship between the variables.

		Estimate	S.E.	C.R.	P	Whether the hypothesis is supported
Food quality	OFD service quality	0.652	0.469	3.545	^***^	
Comments	OFD service quality	0.383	0.170	3.545	^***^	
Kitchen photos	OFD service quality	0.849	0.306	4.781	^***^	
Delivery speed	OFD service quality	0.628	0.253	4.528	^***^	
Packaging	OFD service quality	0.365	0.214	3.460	^***^	
Consumption intention	OFD service quality	0.339	0.197	3.305	^***^	Yes

Note:

*** indicates p < 0.001.

As can be seen from Table 7, the path coefficient of OFD service quality on consumption intention is 0.339, p < 0.001, indicating that OFD service quality has a significant positive impact on customers’ consumption intention, which means that the better OFD service quality of the store, the higher the customers’ consumption intention. Hypothesis 1 is established.

In addition, the path influence coefficients of the five dimensions (Food quality, comments, kitchen photos, delivery speed, and packaging) of OFD service quality are 0.652, 0.383, 0.849, 0.628, 0.339, and 0.365 respectively. All coefficients are significant, indicating that OFD service quality factors can significantly positively promote customers’ consumption intention. Among them, the food quality and the kitchen photos had greater effects.

### 4.3 Hypothesis testing of the calorie labels’ moderating effect

As for the test of the moderating effect of calorie labels in various OFD stores, this article used the “product indicator approach” mentioned by Wen et al. [36] as a reference. At the same time, the item parceling strategy in structural equation modeling proposed by Wu and Wen [37] was studied and discussed.

First, the model of the influence of service quality factors on customers’ consumption intention was parceled. The observed variables for each first-order latent variable in the model were calculated as the mean (Quality_mean, Comments_mean, Photos_mean, Speed_mean, Packaging_mean). Then, these means were used as the observed variables for the original second-order latent variable. Therefore, the second-order model was reduced to a first-order model.

**Fig 3:**
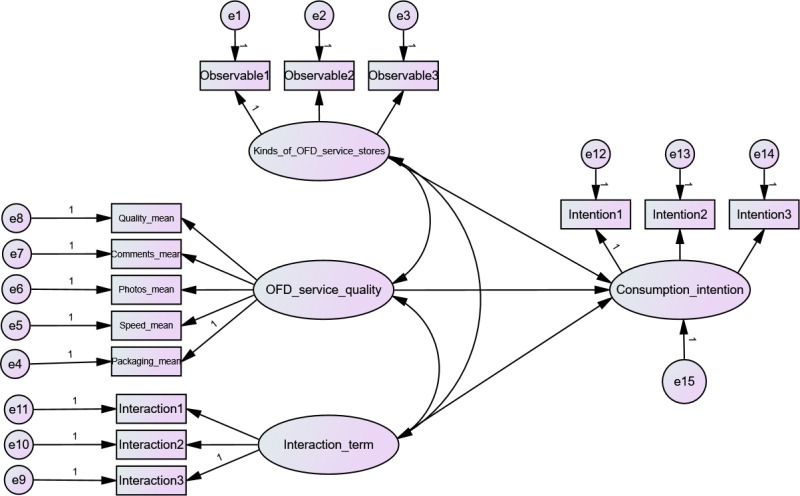
Structural equation model with moderator variables.

Secondly, the product indicator approach was used to create interaction terms between calorie labels of different OFD service stores and the OFD service quality, thereby constructing a structural equation model with moderating effects, which was shown in Fig 3. The influence of the moderator variable on the relationship between the independent and the dependent variables, and its direction (positive or negative), can be accessed via the p-value and path coefficient of their interaction term. If the relationship between the interaction term and the dependent variable is significant, it indicates that the moderating variable has a moderating effect. When the path coefficient is greater than zero, it indicates a positive effect; otherwise, it indicates a negative effect.

**Fig 4:**
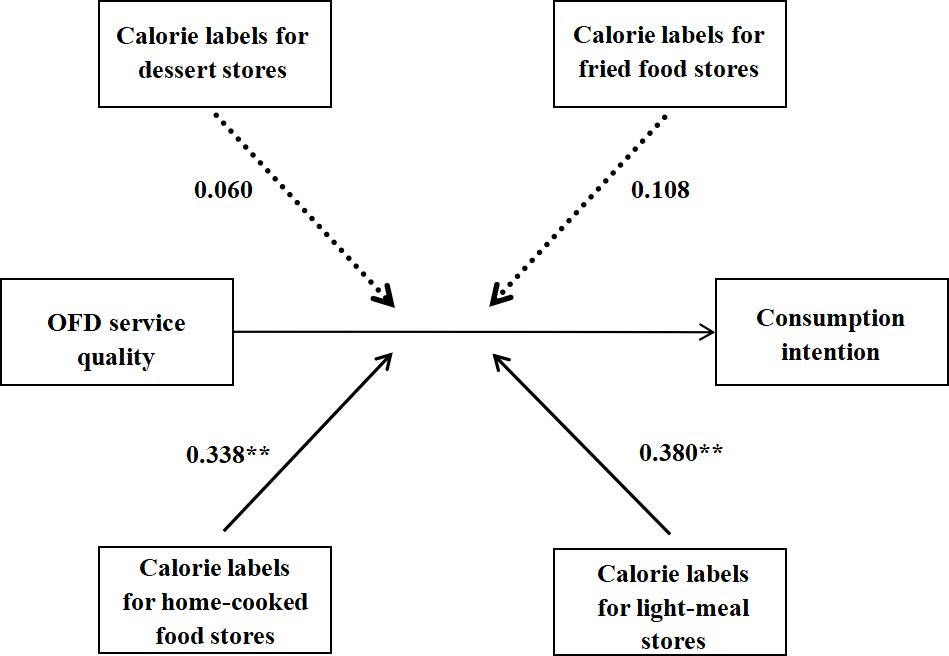
Model standardized path coefficients and significance (Note: ** indicates P < 0.01, the dotted line indicates non-significant.).

Finally, Amos software was used to verify whether calorie labels of different OFD service stores would inhibit or enhance the promotion effect of OFD service quality on consumption intention, which was shown in Fig 4. The path coefficients of interaction items of home-cooked and light-meal food stores are 0.338 and 0.38 respectively. The impact of interaction items on customers’ consumption intention is significant at the level of 1%. It can be seen that for these two types of stores, calorie labels can further promote consumers’ consumption intention under the positive impact of OFD service quality on consumers’ consumption intention. The path coefficients of the interaction terms of dessert shops and fried shops are 0.06 and 0.108 respectively. These coefficients are not significant, indicating that the calorie labels of these two types of stores have no moderating effect under the positive impact of OFD service quality on consumers’ consumption intention., so Hypothesis 2 is not assumed to be valid.

## 5 Conclusion

The research shows that the OFD service quality has a significant positive effect on customers’ consumption intention. The five dimensions of food quality, comments, kitchen photos, delivery speed, and packaging all have a significant positive impact on consumers’ consumption intention. This conclusion can also be confirmed through the research of Wu et al [38]. Their findings revealed that reliability, assurance, maintaining food quality, system operation, traceability, and perceived service value could elevate customer satisfaction and optimize the intention to reuse food delivery services, which is similar to our research. Regarding the moderating effect, calorie labels have a significant positive moderating effect on the impact of OFD service quality on consumers’ consumption for home-cooked stores and light-meal stores, while for dessert and fried food stores, calorie labels have no moderating effect. The conclusion can also be explained by other researches: First of all, consumers who choose light-meal food or home-cooked food usually have a stronger sense of health. In such “health-oriented” situation, adding calorie labels reflects the restaurant’s concern for health, thereby enhancing the positive impact of OFD service quality on consumption. In addition, Camella and Nadine [39] found that calorie labeling increased the likelihood of choosing healthier food and beverage. Home-cooked food and light-meal food are usually less sugar, less oil and less salt, which means that they are healthy. Therefore, calorie labels in home-cooked food and light-meal food stores can increase the likelihood of people’s consumption intention. But when choosing desserts and fried foods, customers are often in a “hedonism-oriented” situation. They pay more attention to hedonic experiences. At this time, calorie labels are likely to be actively ignored by consumers. Emilia et al. [40] analyzed factors that influence appeal of ultra-processed food (such as dessert or fried food), and they found that consumers’ choices for these foods are primarily driven by sensory factors, meaning that these consumers place greater emphasis on taste and enjoyment rather than nutritional content. Therefore, calorie labels in dessert and fried food stores have no moderating effect.

### 5.1 Limitations and future Study

Although this study offers valuable insights, there are some limitations and directions for future researches that should be considered. Firstly, the sample consists of OFD service users from China, which may limit the applicability of the findings to other regions. Cultural differences, varying technology adoption, and other factors may lead to potential variations in results across different countries. Therefore, future research should replicate this model across different countries or cultural contexts to validate the robustness of the findings.

Secondly, this study is limited to four types of OFD service stores: home-cooked food, light-meal food, dessert and fried food. While this classification captures key segments, it does not fully reflect the diversity of the OFD service market. Future studies could include other categories such as beverages, international cuisines and so on to enhance the comprehensiveness of the analysis.

Thirdly, this study only examined a limited number of predictors. Future research may consider incorporating other influential factors, such as price and trust in third-party platforms.

### 5.2 Practical implications

Based on these conclusions, the OFD service industry can be improved based on the following two practical suggestions.

Firstly, the OFD service stores should provide a strict control process from source to dining then to after-sale service. Strengthening strict control over food quality from the source is essential. Higher standards must be applied at every stage, from ingredient procurement to processing, packaging, and delivery, to ensure food safety. After that, the kitchen environment should be disinfected and cleaned every day, and the kitchen photos should be uploaded and updated regularly so that customers can rest assured [41]. At the same time, OFD service stores should speed up delivery and deliver food to customers on time under the premise of ensuring quality [42]. Then, the OFD service packages should be insulated and strong to maximize the good taste [43]. Finally, OFD service stores should improve the quality of after-sales service and solve customers’ problems with patience and enthusiasm, to improve the number of customers’ praise.

Secondly, the OFD service should provide personalized calorie labels. As the customers of light-meal and home-cooked food focus on traditional eating, health, and body management, calorie labels can be indicated in the food section to better help consumers focus on their health. For dessert and fried stores, the food in such stores is mostly high in oil and sugar. If these foods are labeled with calories, customers often feel guilty because they are about to eat high-calorie food. Therefore, OFD service stores can selectively label foods with lower calories to help customers better balance their emotions, thereby improving their willingness to consume.

In conclusion, these suggestions not only can enhance the overall consumer experience, but also facilitate operational improvements within the OFD service industry. Additionally, this study informs platform design, OFD sales, and even public health messaging in the context of online food delivery services.

### 5.3 Theoretical implications

This study contributes to the current researches with several theoretical implications. Firstly, it employs structural equation modeling to explore the impact of OFD service quality on consumption intentions, thereby enriching the existing research framework on electronic commerce and consumer decision-making. The findings also contribute to a theoretical understanding of how the service quality of OFD service platforms influences consumer decision-making processes, offering valuable insights for scholars and practitioners in the fields of electronic commerce, marketing, and consumer behavior.

Secondly, the influence of OFD service mainly focused on price, convenience or service quality in the previous scholars’ studies. However, the results of this research showed that visual trust (such as kitchen photos) can significantly enhance customers’ consumption, providing a new direction for subsequent research.

Thirdly, this study introduces calorie labels as a moderator variable, providing a new perspective for the field of health marketing and highlighting the importance of integrating health awareness into digital marketing strategies.

## Supporting information

S1 Data Dataset. Data from the questionnaire.
(XLSX)

S2 Data Questionnaire. (DOCX)
