# Supplementary material for: Study on the influence of customers’ consumption intention in online food delivery service: Calorie label as the moderator variable
Source: PLoS One. 2025 Jun 26;20(6):e0326617. doi: 10.1371/journal.pone.0326617 (PMC12200688; doi:10.1371/journal.pone.0326617)
Supplement: S2 Data — (DOCX) [file pone.0326617.s002.docx]

**Investigation on influencing factors of consumption intention of online food delivery service customers**

Dear friends,

Thank you for taking the time to participate in this questionnaire regarding the analysis of factors influencing online food delivery service consumers' consumption intentions. There are no standard answers to the questions in the questionnaire. Please answer based on your actual ordering experience and your true thoughts. This survey is anonymous and does not involve any personal privacy. The information you provide will be kept strictly confidential.

Thank you again for your cooperation and participation!

**Part 1: Logical option**

1. *Do you have any experience of ordering food online? [multiple choice]*
2. Yes [If you choose this option, you will continue to fill out the questionnaire]
3. No [Please skip to the end of the questionnaire and submit the questionnaire]

**Part 2: Basic information**

1. *Your gender [multiple choice]*
2. Male
3. Female
4. *Your age [multiple choice]*
5. 18 to 25
6. 25 to 35
7. 35 to 45
8. Over 45
9. *Your occupation [multiple choice]*
10. Student
11. Salaryman
12. Freelance
13. Other
14. *Your monthly income [multiple choice]*
15. 0 to 1000
16. 1000 to 2000
17. 2000 to 4000
18. Over 4000
19. *Your frequency of ordering food online [multiple choice]*
20. Everyday
21. 2 to 5 times a week
22. 2 to 5 times a month
23. Little
24. *Your attention to calorie labels [multiple choice]*
25. Highly concerned
26. Moderately concerned
27. Generally concerned
28. Slightly concerned
29. Unconcerned

**Part 3 Measurement of Research Variables**

This survey was conducted using the Likert Scale. The scoring scale is: 1= strongly disagree; 2= disagree; 3= general agreement; 4= Agree; 5= Strongly agree. Please choose according to your actual feelings.

1. Food quality: [multiple choice]

|  | **1** | **2** | **3** | **4** | **5** | **Reference** |
| --- | --- | --- | --- | --- | --- | --- |
| **Fresh food will increase my consumption intention** |  |  |  |  |  | **Liang (2017)**  **[1]** |
| **Good taste in food will increase my consumption intention** |  |  |  |  |  |  |
| **Nutritious food will increase my consumption intention** |  |  |  |  |  |  |

1. Comments: [multiple choice]

|  | **1** | **2** | **3** | **4** | **5** | **Reference** |
| --- | --- | --- | --- | --- | --- | --- |
| **Before ordering food online, I usually browse comments about the store or the food** |  |  |  |  |  | **Liu (2017)**  **[2]** |
| **Positive comments from other buyers will increase my consumption intention** |  |  |  |  |  |  |
| **Negative comments from other buyers will decrease my consumption intention** |  |  |  |  |  |  |

1. Photos of kitchen: [multiple choice]

|  | **1** | **2** | **3** | **4** | **5** | **Reference** |
| --- | --- | --- | --- | --- | --- | --- |
| **Before ordering food online, I usually browse photos of kitchen about the store** |  |  |  |  |  | **Liu (2017)**  **[2]** |
| **Clear kitchen will increase my consumption intention** |  |  |  |  |  |  |
| **Dirty kitchen will decrease my consumption intention** |  |  |  |  |  |  |

1. Delivery speed: [multiple choice]

|  | **1** | **2** | **3** | **4** | **5** | **Reference** |
| --- | --- | --- | --- | --- | --- | --- |
| **Fast or on-time delivery can increase my consumption intention** |  |  |  |  |  | **Chen (2021)**  **[3]** |
| **The longer the delivery time, the lower my willingness to consume** |  |  |  |  |  |  |
| **Real-time updates on the status of online food delivery service orders can increase my consumption intention** |  |  |  |  |  |  |

1. Packaging: [multiple choice]

|  | **1** | **2** | **3** | **4** | **5** | **Reference** |
| --- | --- | --- | --- | --- | --- | --- |
| **Before ordering food online, I usually browse the food package** |  |  |  |  |  | **Liu (2017)**  **[2]** |
| **Good quality or attractive packaging will increase my consumption intention** |  |  |  |  |  |  |
| **Poor quality or unattractive packaging will decrease my consumption intention** |  |  |  |  |  |  |

1. Consumption intention: [multiple choice]

|  | **1** | **2** | **3** | **4** | **5** | **Reference** |
| --- | --- | --- | --- | --- | --- | --- |
| **I will continue to use  online food delivery services  in the future** |  |  |  |  |  | **Chen (2019)**  **[4]** |
| **I would like to recommend online food delivery service to friends around me** |  |  |  |  |  |  |
| **I am willing to order food online for a long time** |  |  |  |  |  |  |

**Part 4 Measurement of the calorie labels’ influence to different stores**

This survey was conducted using the Likert Scale. The scoring scale is: 1= strongly disagree; 2= disagree; 3= general agreement; 4= Agree; 5= Strongly agree. Please choose according to your actual feelings.

1. Dessert stores: [multiple choice]

|  | **1** | **2** | **3** | **4** | **5** | **Reference** |
| --- | --- | --- | --- | --- | --- | --- |
| **Before I buy this type of food, I want to browse calorie labels** |  |  |  |  |  | **Xia (2017)**  **[5]** |
| **The use of calorie labels for this type of store is necessary** |  |  |  |  |  |  |
| **For this type of store, I would want to buy food with a calorie label** |  |  |  |  |  |  |

1. Fried food stores: [multiple choice]

|  | **1** | **2** | **3** | **4** | **5** | **Reference** |
| --- | --- | --- | --- | --- | --- | --- |
| **Before I buy this type of food, I want to browse calorie labels** |  |  |  |  |  | **Xia (2017)**  **[5]** |
| **The use of calorie labels for this type of store is necessary** |  |  |  |  |  |  |
| **For this type of store, I would want to buy food with a calorie label** |  |  |  |  |  |  |

1. Home-cooked food stores: [multiple choice]

|  | **1** | **2** | **3** | **4** | **5** | **Reference** |
| --- | --- | --- | --- | --- | --- | --- |
| **Before I buy this type of food, I want to browse calorie labels** |  |  |  |  |  | **Xia (2017)**  **[5]** |
| **The use of calorie labels for this type of store is necessary** |  |  |  |  |  |  |
| **For this type of store, I would want to buy food with a calorie label** |  |  |  |  |  |  |

1. Light-meal stores: [multiple choice]

|  | **1** | **2** | **3** | **4** | **5** | **Reference** |
| --- | --- | --- | --- | --- | --- | --- |
| **Before I buy this type of food, I want to browse calorie labels** |  |  |  |  |  | **Xia (2017)**  **[5]** |
| **The use of calorie labels for this type of store is necessary** |  |  |  |  |  |  |
| **For this type of store, I would want to buy food with a calorie label** |  |  |  |  |  |  |

**Reference:**

1. Liang YZ. Research on influencing Factors and Problems of Customer Satisfaction of O2O Takeout Service of Meituan in Harbin. MA thesis. Harbin Institute of Technology. 2020. Available from: https://link.cnki.net/doi/10.27061/d.cnki.ghgdu.2020.005515
2. Liu YQ. Empirical Study on Influential Factors of Customer Behavior Related with Take-away On-line Food Purchasing. MA thesis. Southwest Jiaotong University. 2017. Available from: [在线外卖顾客购买意愿影响因素的实证研究 - 中国知网](https://kns.cnki.net/kcms2/article/abstract?v=fNwONIwGMRLN9nbPZxA-Apq8MFYrIoTrBmgcug6zd1fGvz4KKha3DnePUJanqvqxS__CdnAMIuhMF-4De8LuXxDNcoGhPiCcy9i25fTq3cBZD-jE3oc9Cu9WwSu_GEmt_G0FcXgkT0eF6h-4WljST7SKP_SBbjuqz3js1H3xbKZzGBggEo9gp53-7_grfxmj5LHfxfGdBAU=&uniplatform=NZKPT&language=CHS)
3. Chen M. Research on the Impact of Food Delivery Platform Service Quality on Customer Satisfaction. MA thesis. Qingdao University. 2021. Available from: <https://link.cnki.net/doi/10.27262/d.cnki.gqdau.2021.000736>
4. Chen YK. Study on Influencing Factors of Consumers' Repeat Purchase Intention in Catering takeaway O2O platform. MA thesis. South China Agricultural University. 2019. Available from: <https://link.cnki.net/doi/10.27152/d.cnki.ghanu.2019.001239>
5. Xia YY. A study on the influence of food nutrition labels on consumers' purchasing intention - a case study of health food. MA thesis. Southwest University of Political Science and Law. 2017. Available from: https://d.wanfangdata.com.cn/thesis/ChhUaGVzaXNOZXdTMjAyNDA5MjAxNTE3MjUSCUQwMTIyMTE4MxoIOWxxdGZqamM%3D
